# Effects of Tetrodotoxin on the Mammalian Cardiovascular System

**DOI:** 10.3390/md8030741

**Published:** 2010-03-19

**Authors:** Thomas Zimmer

**Affiliations:** Institute of Physiology II, Friedrich Schiller University, Kollegiengasse 9, 07743 Jena, Germany; E-Mail: thomas.zimmer@mti.uni-jena.de; Tel.: +49-3641-934372; Fax: +49-3641-933202

**Keywords:** Na^+^ channel, TTX sensitivity, cardiac conduction, TTX poisoning

## Abstract

The human genome encodes nine functional voltage-gated Na^+^ channels. Three of them, namely Na_v_1.5, Na_v_1.8, and Na_v_1.9, are resistant to nanomolar concentrations of tetrodotoxin (TTX; IC_50_ ≥ 1 μM). The other isoforms, which are predominantly expressed in the skeletal muscle and nervous system, are highly sensitive to TTX (IC_50_ ~ 10 nM). During the last two decades, it has become evident that in addition to the major cardiac isoform Na_v_1.5, several of those TTX sensitive isoforms are expressed in the mammalian heart. Whereas immunohistochemical and electrophysiological methods demonstrated functional expression in various heart regions, the physiological importance of those isoforms for cardiac excitation in higher mammals is still debated. This review summarizes our knowledge on the systemic cardiovascular effects of TTX in animals and humans, with a special focus on cardiac excitation and performance at lower concentrations of this marine drug. Altogether, these data strongly suggest that TTX sensitive Na^+^ channels, detected more recently in various heart tissues, are not involved in excitation phenomena in the healthy adult heart of higher mammals.

## 1. Introduction

Voltage-gated sodium (Na^+^) channels are responsible for the fast upstroke of action potentials in electrically excitable cells [1]. These channels form heteromultimeric proteins consisting of a large pore-forming *α* subunit and small accessory *β* subunits. Ten different *α* and four *β* subunit isoforms have been cloned from different mammalian tissues [2]. The Na^+^ channel isoform Na_v_1.5, encoded by the *SCN5A* gene, is the predominant *α* subunit in the heart and plays a key role in the excitability of atrial and ventricular cardiomyocytes and in rapid impulse propagation through the specific conduction system [1–5]. Mutations in *SCN5A* can cause a broad variety of pathophysiological phenotypes, such as long QT syndrome type 3 (LQT3), Brugada syndrome (BrS), cardiac conduction disease (CCD), and sick sinus syndrome (SSS) [6,7].

Cardiac Na_v_1.5, as well as Na_v_1.8 and Na_v_1.9 channels, which are both expressed in dorsal root ganglion neurons, are resistant to nanomolar concentrations of the pufferfish poison tetrodotoxin (TTX). The IC_50_ is equal or higher than 1 μM [2,8]. In contrast to the cardiac isoform, neuronal and skeletal muscle Na^+^ channels are highly sensitive towards TTX (IC_50_ ~ 10 nM) [2,8]. The TTX sensitive (TTXs) Na^+^ channels are: (a) Na_v_1.1, Na_v_1.2 and Na_v_1.3, which are highly expressed in the central nervous system, (b) Na_v_1.4, the predominant Na^+^ channel in skeletal muscle, (c) Na_v_1.6, widely expressed in neurons of the central and peripheral nervous system, and (d) Na_v_1.7, which is found in the peripheral nerve system including sympathetic fibers [2].

## 2. TTX Sensitive Na^+^ Channels in the Mammalian Heart—A Brief Summary

During the last two decades, several electrophysiological and biochemical studies have explored the molecular nature of the cardiac Na^+^ current, thereby providing strong evidence in support of the expression of skeletal muscle and neuronal Na^+^ channels in the mammalian myocardium (Table 1, reviewed in [9]). Results were obtained using *in vitro* assays, such as expression pattern analysis by RT-PCR or protein analysis by Western blotting and immunofluorescence. Using electrophysiological techniques on isolated cardiomyocytes, several authors succeeded in demonstrating an often small Na^+^ inward current that could be blocked at nanomolar concentrations of TTX. One study even suggested that the plasma membrane of the middle portion of rabbit ventricular cardiomyocytes contains exclusively TTXs Na^+^ channels [10]. Based on RNA/protein detection methods and on electrophysiological measurements at nanomolar concentrations of TTX (see Table 1), enthusiastic discussions and far-reaching speculations on the function of these channels were published [9,11,12]. However, conflicting results were reported (see Section 5), and moreover, a convincing evidence for an important physiological role of TTXs Na^+^ channels in the adult myocardium of higher mammals was not yet provided.

I thought that if TTXs Na^+^ channels exert direct chronotropic, inotropic, and dromotropic effects in the normal heart, one should observe, first, diminished cardiac conduction and output in animals intoxicated with low, *i.e.*, sub-lethal doses of TTX, and second, impaired cardiac performance in accidentally intoxicated humans. This review summarizes the literature on both aspects. Particular attention is paid to the systemic effect of TTX on cardiovascular functions of higher mammalian animals. The answer to the question, whether or not TTXs Na^+^ channel transcripts and proteins are of any physiological relevance in the normal heart, could help us to better understand basic cardiac excitation phenomena. Moreover, it could be important for the development and application of antiarrhythmic drugs, for the clinical management of cardiac diseases, and even for emergency physicians treating TTX-poisoned patients.

## 3. Systemic Effects of TTX on the Cardiovascular System—Lessons from Animal Experimentations

The first extensive scientific studies on TTX poisoning, published in a western language, appeared at the end of the 19th and beginning of the 20th centuries [25–27]. The authors fed several different mammalian species with pufferfish viscera or, as in most cases, injected purified toxin extracts from hard roe subcutaneously. They determined the minimal lethal dose (MLD) and meticulously described systemic effects and dysfunction of most organs at varying toxin dosages. Later, cardiovascular effects of TTX were intensively studied from the mid 50s to the early 70s, when TTX was commercially available as a highly purified powder and when its chemical structure was identified [28]. In the last 30 to 40 years, research into the systemic effects of TTX in mammals faded again from the scientific spotlight and relatively few articles appeared.

There are some limitations regarding these previous reports that have to be mentioned: Firstly, animal models of cardiac diseases were not considered, but the presumed cardiac TTXs Na^+^ channels could play a role in the diseased heart. In the ischemic and reperfused myocardium, a TTXs persistent Na^+^ current component (I_Na(P)_) is characteristically increased, which may trigger afterdepolarizations and life-threatening arrhythmias (reviewed in [29]). The molecular nature of this pathologically enhanced I_Na(P)_ is not entirely clear [29]. This current fraction could be either caused by a specific gating mode of Na_v_1.5 [30,31] or by non-inactivating neuronal Na^+^ channels (reviewed in [9]). Notably, Na_v_1.1 was upregulated in a rat infarct model and concomitantly prolonged action potentials could be shortened by 100 nM TTX [22]. Furthermore, it is possible that upregulation or modulation of TTXs Na^+^ channels preserves cardiac conduction under hypoxic/ischemic conditions, because steady-state inactivation in TTXs Na^+^ channels is positively shifted, compared to Na_v_1.5 [9,32]. Consequently, TTXs Na^+^ channels would be available in a respectively depolarized myocardium, thereby safeguarding cardiac conduction [9,32]. Secondly, only adult animals were used, but TTXs Na^+^ channels may be subject to developmental regulation in the heart [5,13,23,33,34]. Thirdly, there were no systematic studies on possible gender differences [5]. Several previous studies included both male and female animals. A significant difference was not reported [35–40]. Fourthly, mice were often used to estimate the lethality of a TTX solution, but the reaction of their cardiovascular system to intoxication was not or rarely investigated. In contrast to higher mammals, mice may require a high density of TTXs Na^+^ channels in their sinus node and ventricles to maintain the extremely high heart rate and the resulting fast conduction. Fifthly and most importantly, effective toxin concentrations, as well as the distribution and fate of TTX in the body, often remained uncertain. The toxin was given orally, intravenously (i.v.), subcutaneously (s.c.), intraarterially (i.a.), or intraperitoneally (i.p.). Blood or urine levels of TTX were not systematically investigated. It is known that TTX distributes nearly everywhere in the body fluid [41], but significant concentration differences may exist between the organs. In one study, highest concentrations were found in rat kidney and whole heart, lowest concentrations in brain and blood [41,42]. The half-time of disappearance of the toxin after subcutaneous injection varied from 30 minutes to four hours, depending on the organ [42].

Despite these uncertainties, the systemic application of TTX seems to be superior to most of the more recently applied *in vitro* approaches (Table 1), because TTX acts on Na^+^ channels in their natural environment and at physiologic membrane potentials. For example, TTXs Na^+^ channels might be detectable *in vitro*, but they could be actually unavailable for excitation *in vivo*. Thus, they could be physiologically irrelevant for normal cardiac function. Concerning the effective toxin concentration, one can assume that TTX doses used in previous studies were high enough to block the vast majority of the presumed functional TTXs Na^+^ channels in the adult mammalian heart: Firstly, injecting one MLD, equivalent to 5–10 μg/kg (see Table 2), and assuming a uniform distribution in the extracellular space, the effective TTX concentration should be nearly 80–160 nM [37]. Toxin dilution on the way to the heart should be much less pronounced, when injecting the drug bolus i.v. within a few seconds [43]. For example, application of 0.5–1.0 MLD into the jugular vein within seconds could result in such a high TTX concentration in the heart that already blocks a portion of Na_v_1.5 channels. Secondly, TTX at large sub-lethal doses resulted in severe impairment of many neurological/neuromuscular functions that are all controlled by TTXs Na^+^ channels. Symptoms are urination, hypersalivation, retching, vomiting, diarrhoea, diminution or absence of reflexes, skeletal muscle fasciculations, lethargy, ataxia, ascending progressive paralysis, respiratory pattern changes, and dyspnoea [35,36,41,44]. Death is due to a rather complex action of TTX on the respiratory system, involving blockade of the phrenic nerve, the diaphragm and neurons in the central respiratory network [26,27,36,41,45].

Most, if not all, investigators excluded direct TTX-specific cardiac irregularities. Their studies provided overwhelming evidence that the heart belongs to the very few organs that remain nearly unaffected, even at large sub-lethal or lethal TTX doses. Note that intoxication often required artificial ventilation, but the heart continued beating regularly [37–41,44,46,47]. Nevertheless, TTX exerts pronounced depressive effects on the circulatory system (Table 2). Cardiovascular reactions of intoxicated animals are a reduced blood pressure (3.1), bradycardia (3.2), and in few cases, reduced ventricular force and stroke volume (3.3). Conduction disturbances occurred rarely and were often characteristic of a severe TTX intoxication (3.4). In the following sections, the mechanisms for these cardiovascular reactions are summarized and discussed.

### 3.1. Hypotension

Reduction in arterial blood pressure can result from a reduced total peripheral resistance (TPR) and/or a reduced cardiac output. Already in 1890, Takahashi and Inoko concluded that the heart does not contribute to the TTX-mediated blood pressure reduction [26]. The authors explained the hypotensive action of the toxin exclusively with a reduced vasomotor tone, resulting in a significant TPR reduction. Compression of the abdominal aorta or volume expansion instantaneously normalized a markedly lowered carotid blood pressure and a slightly lowered pulse rate in TTX-poisoned cats and dogs [26]. A direct negative inotropic or chronotropic action of TTX in intoxicated animals was excluded also in most if not all subsequent studies. For example, Cheng and colleagues [43,46] found that the blood flow in the ascending aorta was not measurably altered when injecting a dose of 0.25 MLD into right external jugular vein (Table 2), demonstrating that the simultaneously observed arterial hypotension was caused by peripheral vasodilatation only.

The mechanisms behind the TTX-mediated vasodilatation were investigated by several groups. The significance of each mechanism, however, was discussed controversially [39,41]. Firstly, TTX could act on medullary structures known to be involved in cardiovascular control after the toxin crossed the blood-brain barrier [26,27,35,36,41,45]. Hypotension occurred nearly instantaneously when low doses of TTX were injected into the brain or carotid artery of cats and rats [35,48]. In another study, a fast vasodepressive response and a delayed but significant bradycardia was observed when 12.5–25 pmol TTX were injected into the cat nucleus reticularis lateralis (NRL), a ventromedullary vasopressive region [40]. Microinjection of the same TTX dose into the nucleus tractus solitarii (NTS), a well known vasodepressive and cardio-inhibitory area, caused an increase in blood pressure. Secondly, vasodilatation resulted mainly from a conduction block in sympathetic fibers [27,35,37,39,41,43,46]. Thirdly, low doses of TTX may directly affect vascular smooth muscles [39]. Several authors, however, did not observe any effect of TTX on smooth muscle tone [27,37,43].

### 3.2. Bradycardia

Slowing of the heart rate may also contribute to hypotension. However, administration of small sub-lethal doses produced hypotension only, and apparent changes of heart rate were rarely seen. TTX in doses of 0.1–0.3 MLD, corresponding to about 16–47 nM in the whole body’s extracellular fluid, did not cause bradycardia in the cat, despite a prompt and marked fall in blood pressure [37–39,49]. For example, Kao *et al.* demonstrated that the carotid blood pressure in cats can be reduced by i.v. injection of 2–3 μg/kg from 140/110 to 80/50 within two to three minutes, without affecting heart rate [41]. In some studies, even lethal doses of 7–10 μg/kg did not significantly alter heart rate in cats [38]. When heart rate reduction was measured, it often occurred subsequent to the onset of hypotension [38,40,45,46]. Severely intoxicated guinea pigs (15 μg/kg, i.p.) showed no signs of ECG alterations shortly before respiratory failure and death, despite a substantial drop in blood pressure [45]. In pithed rats, heart rate fell about 10% after injection of 1–2 MLD, but also soon regained the pre-injection state, in contrast to the long-lasting blood pressure reduction [43]. In another study, a 30–40% reduction in heart rate was observed in intoxicated rats (20 μg/kg). A few minutes after artificial respiration was commenced heart rate increased by 20%, but blood pressure further declined [48]. Altogether, these data indicate that bradycardia is not the primary cause of hypotension, although it may contribute to some extent to a reduced arterial blood pressure, in particular in severe cases of TTX intoxication.

Concerning the mechanism behind the observed bradycardic effect, one can exclude a primary chronotropic effect *via* blockade of cardiac TTXs Na^+^ channels: Firstly, direct application of a highly concentrated pufferfish solution or a toxin powder on the sinus node had no effect on the heart rate [27]. Secondly, Tomlinson and James [47] injected a volume of 2 ml of a 310 nM solution rapidly into the sinus node artery of dogs in order to avoid profound extracardiac effects of systemically applied TTX. The authors noticed that heart rate and rhythm remained constant. Only a 10-fold higher concentration caused a negative chronotropic effect (see below). Thirdly, sympathetic denervation of the cat heart resulted in a fall in heart rate to ~42–71%, but the subsequent application of a relatively high toxin dose (15 μg/kg) did not further decrease the heart rate [37]. Fourthly, the same lethal TTX dose of 15 μg/kg was rapidly injected as a bolus intraperitoneally into guinea pigs [45]. The authors noticed a decline in blood pressure, but even several minutes after intoxication, a regular heart rate and no change of the ECG wave form. ECG alterations indicative of paroxysmal ventricular tachycardia, sinus bradycardia and atrioventricular (AV) block emerged only after a complete cessation of respiratory movement and the substantial drop in arterial O_2_ (see also Section 4). Fifthly, TTXs Na^+^ currents do not contribute to normal automaticity in isolated adult sinus node cells, as recently shown by Protas and co-workers [34]. The authors demonstrated that Na^+^ current density in the canine sinus node decreases with age and that TTXs Na^+^ channels are not available at physiological potentials [34]. Application of 100 nM TTX did neither change cycle length nor action potential parameters of adult sinus node cells. Consequently, TTXs Na^+^ channels are present in the sinus node, but they do not exert a physiological function under normal conditions [34]. This conclusion is in agreement with previous data [50].

The transient or permanent reduction of the heart rate is most likely the result of a complex systemic reaction to TTX intoxication. There are several parameters that interfere with a normal heart beat in intoxicated mammals, like (a) a diminished pressoreflex despite hypotension, due to blockade of respective afferent and efferent nerve fibers, (b) a reduced venous return, due to massive vasodilatation and hypotension [39], (c) uncertain effects of respiratory irregularities on medullary cardiovascular centers normally involved in respiratory sinus arrhythmia, (d) direct effects of TTX on medullary neurons [26,35,40,45], or (e) block of sympathetic nerve fibers [37]. In particular the latter effect seems to play a predominant role in TTX-induced bradycardia [37], because in isolated sympathetic nerve-right atrium preparations, TTX at low concentrations (30–60 nM) abolished positive inotropic and chronotropic responses to nerve stimulation without any effect on spontaneous rate or force.

At very high TTX doses of at least 1 MLD, the toxin has a direct cardiac effect [26,27,43], most likely *via* blocking an increasingly number of Na_v_1.5 channels, leading to sinus bradycardia and conduction disturbances (see Section 3.4). This conclusion is supported by the following observations. Firstly, bradycardia was induced only when injecting a TTX solution of 3 μM into the canine sinus node artery (see above) [47]. Similar data at 3 μM TTX were observed using isolated rabbit atrial muscle strips including the sinus node [51]. Notably, sinus node action potentials were retained even at a concentration of up to 30 μM, which is far in excess of what is necessary to block conduction of nerve and skeletal muscle [51]. Secondly, very similar symptoms, *i.e.*, sinus bradycardia and/or sinoatrial block, are observed in familial SSS, a well-recognised loss-of-function *SCN5A* channelopathy [7]. Recently, Verkerk and co-workers even succeeded in demonstrating a large Na^+^ inward current in human sinoatrial node cells at resting potentials negative to −60 mV [52]. Although the molecular nature of this current could not be elucidated in detail, the pronounced hyperpolarized closed-state inactivation clearly points to Na_v_1.5 channels. And thirdly, heterozygous *Scn5a*^+/−^ mice showed depressed heart rates and occasionally sinoatrial block [53], implicating an important role of the normal cardiac Na^+^ channel, Na_v_1.5, for pacemaker rates and sinus node conduction.

### 3.3. Stoke volume reduction

A reduced contractile force at low TTX concentrations has been suggested for Langendorff-perfused mouse and guinea pig hearts [11]. Consequently, a resulting decline in stroke volume should be visible in intoxicated animals, and this decline should contribute to hypotension.

However, unchanged stroke volumes were observed in independent studies, when sub-lethal or, depending on the application method, even lethal TTX doses were applied. For example, an unchanged blood flow in the ascending aorta of rats was observed, when injecting a sub-lethal dose of 2.5 μg/kg into the right external jugular vein. At this dose, arterial blood pressure was reduced from 120–150 to 40–55 mmHg [43]. In a similar study, injection of 5 μg/kg caused a rapid fall in blood pressure at an initially unchanged heart rate and stroke volume [46]. Furthermore, slow intravenous TTX injections of 9.3 μg/kg, a lethal dose for dogs, did not alter the cardiac index until respiratory arrest [44]. At apnoea, the authors reported blood pressure reduction and decline in heart rate at an unchanged stroke volume. Using isolated perfused rat hearts, the TTX-containing blue-ringed octopus venom caused even a marked increase in ventricular contraction tension [48], although this effect could be caused by other compounds in the toxin extract.

In some studies, a reduced contractile force was observed upon TTX intoxication [35–37,43]. The apparent negative inotropic action of TTX was discussed as the result of three different mechanisms: (a) blockade of excitability of central and/or peripheral nerve fibres at nanomolar concentrations, preventing neurogenic catecholamine release [35–37,41,43], (b) a direct negative inotropic effect occurring at micromolar concentrations in association with the reduction of the maximum rate of depolarization of the ventricular action potential, an effect that was not seen at 30 to 120 nM TTX [43,54–56], and (c) reduced cardiac output when marked peripheral vasodilatation caused a reduction in venous return [39,46]. The latter mechanism is probably the most important one, because hypotension occurred often sooner than cardiac depression [38]. Moreover, compression of the abdominal aorta as well as volume expansion normalized arterial blood pressure and cardiac performance instantaneously [26,27,39,41].

### 3.4. Cardiac conduction disturbances

ECG alterations indicative of cardiac conduction abnormalities were observed rarely at sub-lethal doses of TTX (Table 2). Only slight PR prolongations were observed in cats and dogs when injecting 2.5–10 μg/kg [37,38]. In other studies, ECG alterations were not seen in the same species at similar toxin concentrations [35,36,43,49]. In guinea pigs, the ECG waveform remained unchanged even minutes after injecting a high lethal toxin dose (15 μg/kg) [45].

Already in their early reports on the systemic effect of TTX, the Japanese researchers noticed conduction abnormalities in severely intoxicated animals, particularly after the cessation of respiration [26,27]. Severe TTX intoxication, which is typically characterized by cyanosis, areflexia, apnoea and hypotension, often triggered second or third degree AV block. In many intoxicated animals, ventricular contractions appeared regular, but at a lower frequency than those of the atria. Subsequent to complete AV block, ventricles stopped beating, but atria not. In one case, Takahashi and Inoko found atrial contractions that persisted even after the onset of rigor mortis [26]. Although it seems that these previous reports are a little antiquated, basically similar results were observed tens of years later using advanced techniques [37,43,45,57]. High lethal TTX doses (1–4 MLD) often caused conduction irregularities such as lengthening of the AV interval, true AV dissociation, bundle brunch block, ventricular flutter or fibrillation. Ventricular asystole was noticed when rapidly injecting a dose of 8 MLD in rats [43].

Conduction disturbances in severely intoxicated animals were most likely due to two mechanisms: (a) blockade of a significant portion of cardiac Na_v_1.5 channels, and (b) insufficient oxygen supply. The first conclusion is supported by the observation that conduction abnormalities were often associated with *SCN5A* channelopathies, like isolated CCD or LQT3 [6]. Moreover, Scn5a^+/−^ mice showed impaired AV conduction [58]. The second conclusion is supported by the fact that AV conduction block or paroxysmal ventricular tachycardia was observed subsequent to respiratory arrest and to the onset of hypoxia [26,45].

According to a previous report [17], one should expect that the Purkinje fibers are the cardiac structures that respond most rapidly to TTX treatment with a diminished function. However, it is interesting to note that the AV node is obviously most susceptible to TTX. Yoo and colleagues precisely studied Na_v_1.5 expression in different regions of the AV node [59]. Na_v_1.5 labeling was absent in the open node, but present at a relatively low level in the pathways into the open node (inferior nodal extension and transitional zone). This suggests that the reduced expression of Na_v_1.5 already diminishes the safety factor for conduction, and consequently, TTX selectively aggravates conduction in this AV nodal region.

Other ECG changes were rarely reported. Some authors noticed T-wave alterations, like a non-specific T wave reversal [38], the disappearance of the T wave [27], or an increase in amplitude [49]. Reasons for these phenomena are unknown. Kao and Fuhrman suggested an involvement of the adrenal medulla in intoxicated animals, and consequently, an indirect effect on the heart of circulating catecholamines [49]. In another study, a decrease in P wave at a relatively large sub-lethal dose of 5 μg/kg was found in dogs [38].

## 4. Human Tetrodotoxication

### 4.1. Epidemiology, symptoms, and prognosis

TTX is one of the most potent, non-protein poisons known to man. It has been detected not only in many pufferfish species, but also in a wide variety of other animals, including the blue-ringed octopus, newts, gobies, frogs, worms, starfish, horseshoe crab, gastropods, and in several marine microorganisms, that produce and accumulate the toxin [60,61]. In the pufferfish body, TTX concentration and distribution depend on the species. Ovary, liver and skin usually contain highest TTX concentrations [60].

Despite, or maybe even because of its severe toxicity, pufferfish is a speciality in Japan. The fish, also known as “fugu”, is normally prepared by licensed puffer cooks, making the (expensive!) meals a culinary adventure, rather than a hazardous Russian roulette. Pufferfish poisoning or tetrodotoxication is, however, still one of the most common food poisonings along the coast of Asia. Ingestion of the toxic fish can be intentional or inadvertent. In Japan, it may happen by eating homemade liver dishes prepared from self-caught pufferfishes. In Bangladesh, several outbreaks of TTX poisoning with dozens of victims were reported, which occurred after pufferfishes (called “potka” fish) were available at local markets at a very cheap price [62–64]. Emergency physicians in Southern Europe should also be aware of the typical intoxication symptoms. Probably due to climate change and rising water temperatures, the toxin has approached the Portuguese coast, and caused recently the first case of tetrodotoxication in Europe (intoxication by a trumpet shellfish species) [65,66].

There is a long history of TTX intoxication. The first Europeans severely intoxicated were Captain Cook and his naturalists, J.R. and G. Forster. They enjoyed a pufferfish dinner, survived, and precisely described their symptoms [67]. Many case reports were known until 1941, when Fukuda and Tani provided a clinical grading system for TTX poisoning [61,68]. This four-degree classification is based on symptoms and stages of progression, and is still of clinical value (Table 3). The degree of intoxication actually depends on three factors: the TTX amount ingested, the time until admission to the emergency department, and pre-existing diseases (see below). First-degree and second-degree cases are relatively mild cases of tetrodotoxication (Table 3). There is a diminution or loss of some neuromuscular and neurological functions, but reflexes are still intact. The third degree is characterized by more severe disturbances, like ataxia, widespread paralysis, pronounced hyporeflexia, drop in blood pressure, fixed/dilated pupils, cyanosis, and respiratory failure (e.g., dyspnoea, decreased vital capacity or lower forced expiratory volume). Hypothermia also develops, when skeletal muscle contractions and conduction in nerve fibers are gradually blocked. Fourth-degree cases are severely intoxicated victims presenting with cessation of respiration, decreased arterial O_2_, unconsciousness, bradycardia, and hypotension. Severe cardiac manifestations, like conduction block or ventricular asystole, occurred when extremely high levels of TTX were ingested and as a late sign in fourthdegree intoxicated patients [68–70].

Tetrodotoxication can be rapidly fatal, and antidotes do not exist. Death may occur in as little as 17 minutes after ingesting the toxin [71,72]. The human killing dose is assumed to be 1–2 mg [61,72,73]. Treatment is entirely supportive and may involve mechanical ventilation for oxygen supply, normal saline infusion for distending the intravascular volume, gastric emptying procedures, application of activated charcoal, atropinization, or treatment with dopamine. Cholinesterase inhibitors and other drugs have been suggested, but not tested adequately [69,70,74]. Prognosis is good if the patient arrives at the emergency department conscious and prior to respiratory arrest, and if the patient survives the first 24 hours. Symptoms usually resolve over a period of 24 hours to five days [64,65,75–77]. Early diagnosis and prompt clinical management essentially contribute to low mortality rates. In Japan, the fatality rate fell from 80% at the beginning of the last century, with more than 100 deaths per year, to about 6% in the 90s [41,60,61]. In other Asian countries, mortality rates between 2–22% were reported recently [62–64,75].

In contrast to the *in vitro* assays (see 2) and *in vivo* animal experimentations (see 3), when crystalline TTX was used, human tetrodotoxication occurs by ingesting TTX-containing food. It is possible that this crude or combined TTX is more easily absorbed in the digestive tract. A respective crude TTX dose of as little as 20 μg/kg can be lethal for humans. However, feeding cats with a 10-fold higher dose of crystalline TTX (200 μg/kg) did not kill them, although an i.v. or i.p. injection of 10 μg/kg was lethal [35]. Furthermore, it is possible that the toxin-containing food exerts slightly different effects in the human body, when compared to those observed after injecting a highly purified TTX solution into animals. Some marine species may produce and accumulate also smaller quantities of TTX analogs, or possibly even other toxic compounds, so that the symptoms may not always be solely due to authentic TTX [66].

### 4.2. Correlation between blood TTX levels and intoxication symptoms

**Figure 1:**
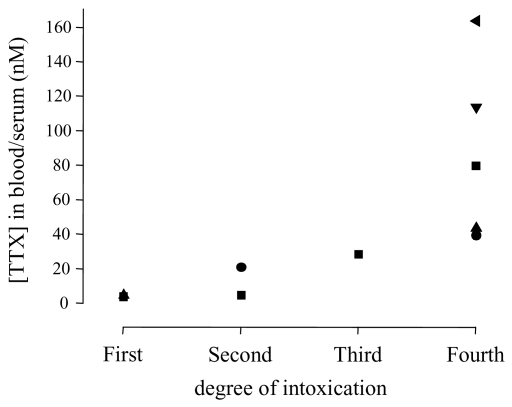
TTX concentrations in blood or serum samples of intoxicated patients. The degree of intoxication was either reported by the authors or assessed according to documented symptoms (for individual data points see Table 4 and the respective references). The lowest and highest concentration reported in [62] was assigned to the first and fourth stage, respectively. Patients 4, 2, 3, and 1, previously reported in [76,81] were assigned to the first, second, third, and fourth stage, respectively. A value of 80 nM was assumed for a severely intoxicated patient whose serum and blood levels were about 83 and 77 nM, respectively [65,66].

Electrophysiological recordings demonstrated an IC_50_ of about 10 nM for TTXs Na^+^ channels [2]. In order to compare this *in vitro* TTX sensitivity with blood levels in patients, and to correlate the *in vivo* TTX concentrations with the symptoms, all currently available blood concentrations were plotted against the intoxication grade of the corresponding patients (Figure 1; for values see Table 4). In less severely intoxicated patients (first and second degree), blood levels were similar to or lower than the IC_50_ of TTXs Na^+^ channels. In severe cases of intoxication (fourth degree in Figure 1), TTX concentrations of 40–164 nM were observed. The calculated average concentration of 88 ± 22 nM (n = 5) would be indeed high enough to block the vast majority of TTXs Na^+^ channels in the body.

Unfortunately, a precise correlation between the biological activity of an ingested TTX bolus and the clinical symptoms is currently difficult to establish, because blood concentrations in victims were rarely determined. It is likely that the effective TTX concentration in the heart is higher than the values reported. All TTX concentrations were determined from “spot” samples taken during the first day of hospitalization. Peak concentrations were probably missed in some cases. Moreover, serum levels may not necessarily indicate the effective concentration in an organ. TTX is rapidly absorbed in the human digestive tract, but it remains in the serum compartment only for a relatively short period (less than 24 h). From day two of hospitalization, TTX serum levels were below the detection limit in severe intoxicated patients [78,79], but TTX appeared in the urine until day four [78]. During this time, intoxication symptoms persisted and mechanical ventilation was still required [79]. It seems that TTX is rapidly eliminated from the serum not only by excretion in the kidney, but also *via* an efficient binding to the TTX receptors. Thus, the toxin may accumulate in an excitable tissue. As suggested by O’Leary and colleagues [80], elimination of TTX from the body requires unbinding from Na^+^ channels and redistribution to the serum. This could be the rate-limiting step in the elimination of TTX from the body. The idea that TTX accumulates in organs with TTX binding sites is supported by previous work of Ogura who detected high TTX levels in whole heart and kidney (excretion), and lowest concentrations in brain and blood [41,42]. Low levels in the brain suggest a limited ability of TTX to cross the human blood-brain barrier. In this context it is interesting to note that victims often remain conscious, but cannot speak, move or breathe.

### 4.3. Cardiac excitation and performance in intoxicated patients

Cardiac dysrhythmias, like AV node conduction abnormalities, bundle-brunch block, tachycardia, or even ventricular stoppage were previously reported to occur in some severely intoxicated patients [68,69]. Because a lethal dose of about 2 mg TTX is unlikely to result in serum concentrations significantly higher than 200 nM, blocking cardiac TTXs Na^+^ channels, rather than blocking Na_v_1.5, could contribute to these cardiac dysrhythmias. However, cardiac excitation abnormalities may not causally depend on a direct action of TTX on cardiomyocytes, but mainly on oxygen limitation resulting from respiratory arrest. If the latter assumption is true, cardiac dysrhythmias in severely intoxicated victims should have been observed rarely during the last 25 years, because of the high standard of modern medicine and the prompt availability of a mechanical ventilation device when respiratory arrest is imminent.

To prove this assumption, a literature screening for reports on outbreaks and cases of tetrodotoxication between 1983 and 2009 was performed (Table 4). Particular attention was paid to cardiovascular effects in victims. Table 4 represents a summary of several representative reports, but not a complete catalog of all cases of tetrodotoxication. In those 20 studies, more than 500 cases of tetrodotoxication were reported. Notably, all patients were admitted to an emergency department or an intensive care unit. However, the cardiovascular status, including blood pressure or ECG, was often not explicitly mentioned, despite an accurate description of neurological and neuromuscular symptoms.

The overall mortality rate during this 27-year period was about 6.6% (34 fatal cases). Hypotension was reported in 22 cases (4.2%). Blood pressure reduction was usually accompanied by rather severe other symptoms, like respiratory arrest or block of nerve conduction. Among fourth-degree cases, only 20.6% of the patients developed hypotension, as documented in [75]. Sinus bradycardia was mentioned only in seven cases, and two of those patients had a pre-existing chronic disease (diabetes mellitus). Surprisingly, AV block was diagnosed only in one patient, who experienced cardiac arrest before arriving at the emergency department and who also had diabetes mellitus [76]. No other pathological ECG signs, like bundle brunch block or ventricular arrhythmias, were reported (Table 4). Even severely intoxicated patients, *i.e.*, fourth-degree victims, presented with a stable cardiovascular status, when they were successfully resuscitated or when oxygen supply was rapidly provided by artificial ventilation [65,89]. In a recent case report, the patient’s serum contained nearly 80 nM TTX, which was shown to be high enough to completely block nerve conduction [65]. At the same time, heart rate and blood pressure were normal, and cardiac conduction disturbances were not observed [65]. The authors concluded that cardiac arrhythmias or cardiac arrest, as observed for example in [85], were due to hypoxemia secondary to respiratory muscle paralysis [65]. Higher blood concentrations of 114 nM were observed in another patient that developed bradycardia and hypotension, but no cardiac conduction abnormalities were noted [78]. More severe symptoms and poor prognosis were reported for victims with a pre-existing disease, in particular for diabetes mellitus patients [76,83]. The reasons are unknown, but there could be a synergistic effect of diabetic neuropathy and TTXs Na^+^ channel blockade on nerve and skeletal muscle excitability [76].

It is interesting to note that cardiovascular manifestations, like hypotension and sinus bradycardia, cannot be considered as general accompanying symptoms in severely intoxicated and mechanically ventilated patients [65,88,89]. Some patients even presented with hypertension [83]. If TTXs Na^+^ channels increase the spontaneous heart rate and improve the contractile force under normal physiological conditions, their blockade by TTX would result in a decreased cardiac output. In order to maintain (or increase) the arterial blood pressure, such a cardio-depressive effect must be counteracted by an increased vasomotor tone. This scenario is very unlikely to occur when conduction in sympathetic fibers is partially or completely blocked. The TPR in TTX-poisoned patients was not yet determined. However, TTX should produce vasodilatation instead of vasoconstriction, and hypotension can be sufficiently explained by a reduced vasomotor tone (see 3).

The heart beat was regular, also when sinus bradycardia was noted [70,87]. In another study, an intermittent sinus bradycardia was reported [86]. The occurrence of cardiac dysautonomia, *i.e.*, transient increases and decreases in heart rate, did not correlate with the TTX levels [86]. As discussed by the authors, the phenomenon of intermittent sinus bradycardia is likely due to TTX-mediated imbalances between the parasympathetic and sympathetic nervous system [86].

In conclusion, severe neurological and neuromuscular symptoms in third-degree and fourth-degree intoxicated victims strongly suggest that a significant portion of functional TTXs Na^+^ channels was blocked. Except for one patient, who experienced cardiac arrest before admission to the emergency department, ECG abnormalities, indicative of cardiac arrhythmias like SA block, AV block, bundle brunch block or ventricular tachycardia/fibrillation, were not reported to occur during treatment of the patients in an emergency department or intensive care unit. This strongly suggests that cardiac excitation is not significantly impaired in intoxicated victims with relatively high TTX blood levels, as long as sufficient oxygen is provided.

## 5. Conclusions

This review on the systemic effects of TTX is not the first account suggesting that Na_v_1.5 is the only functional voltage-gated Na^+^ channel in the adult, non-diseased myocardium of higher mammals. The clinical data are in close agreement with results from animal experiments, a conclusion that was already drawn as early as in the 19th century [26]. Altogether, these data argue against a physiological function of cardiac TTXs Na^+^ channels in higher mammals. This conclusion is strongly supported also by several biochemical and electrophysiological studies, demonstrating (a) Na_v_1.5 in all plasma membrane regions of ventricular cardiomyocytes [18,23,91], (b) only minute quantities of transcripts for TTXs Na^+^ channels in the whole human heart [4,5], (c) inactivated TTXs Na^+^ channels in the sinus node at normal membrane potentials [34,50], (d) unchanged conduction in Purkinje fibers at low TTX concentrations [92,93], and (e) no contribution of TTXs Na^+^ channels to excitation-contraction coupling [94].

## Figures and Tables

**Table 1:** Suggested functions of TTXs Na^+^ channels in the mammalian myocardium. A detailed overview on tissue distribution and detection methods for the individual TTXs Na^+^ channels was given by Haufe *et al.* [9].

Study	Results and suggested function of TTXs Na^+^ channels	Species
	***Sinus node automaticity and control of heart rate***	
[13,14]	Na_v_1.1 transcripts and TTXs currents (IC_50_ ~ 26 nM) in newborn (but not adult) sinus node cells, suggesting that, depending on age, TTXs Na^+^ channels contribute to slow diastolic depolarization.	Rabbit
[12]	Reduction in spontaneous heart rate by blocking Na_v_1.1/Na_v_1.3 at 100 nM TTX; important contribution of TTXs Na^+^ channels to sinus node automaticity and rhythm, suggesting a possible contribution to SSS in man.	Mouse
[15]	Slowing of pacemaking in intact sinus node preparations and isolated cells at 10 and 100 nM TTX, slowing of both pacemaking and sinus node conduction at 1–30 μM TTX.	Mouse

	***Efficient EC coupling and increased cardiac contractility***	
[11]	Reduction of left ventricular function at 100 and 200 nM TTX, suggesting an unexpected role of brain-type Na^+^ channels in excitation-contraction coupling.	Mouse, guinea pig
[16]	Localization of brain-type Na^+^ channels and two *β* subunits in transverse tubules of myocytes, suggesting AP propagation from the cell surface into the interior by defined *α*/*β*-channel complexes.	Mouse

	***Purkinje fibers: Efficient cardiac conduction and AP prolongation***	
[17]	Shortening of AP duration, but not of the maximum rate of rise, at low TTX (≥33 nM).	Dog
[18]	Higher transcript levels and TTXs currents in Purkinje fibers (35 and 22%), when compared to ventricular myocytes (<20 and 10%, respectively).	Dog
[19]	Expression of Na_v_1.4 in cardiac Purkinje myocytes (PCR, immunofluorescence).	Dog

	***Other reports***	
[20]	Detection of Na_v_1.1 transcripts in the heart.	Rat
[21]	Cardiac Na^+^ channels are composed of either Na_v_1.1 or Na_v_1.5, and both associate with *β*1 and *β*2.	Mouse, rat
[22]	Up-regulation of Na_v_1.1 and increased TTXs Na^+^ current in the postinfarction remodeled myocardium.	Rat
[5,23]	Large transcript pool in whole hearts (30–40%), smaller TTXs Na^+^ currents in ventricular myocytes (8%) of mice (not observed in pigs and humans).	Mouse
[10]	Middle region of ventricular myocytes contains only TTXs Na^+^ channels, that can be blocked by 50 nM TTX.	Rabbit
[24]	Prolongation of the cycle length of the spontaneous pacemaker activity at 100 nM TTX by 22% and 53% in sinoatrial and atrioventricular node preparations, respectively.	Mouse

**Table 2:** *In vivo* effect of TTX on the cardiovascular system of various mammalian species. Only studies with a strong focus on the cardiovascular system were included. The minimal lethal dosages (MLD), applied i.p. or i.v., were 2.7–10 μg/kg for rats [41,43], 4.5 μg/kg for guinea pigs [41], 8–10 μg/kg for mice, rabbits, dogs, and cats [35,36,41,49]. Neuromuscular function was already severely affected at sub-lethal dosages. Given orally, the MLD in cats, but not in humans (see Section 4), was at least 20-times higher [35].

Study	Species	TTX app-lication	TTX dose	Cardiovascular effects
[26]	dog, cat, rabbit, rat	s.c.	Tetrodon hard roe extracts	mild intoxication: ataxia and paresis at normal heart function and blood pressure severe intoxication: cyanosis, areflexia, respiratory arrest, hypotension, bradycardia, AV block
[27]	rabbit bufo	i.v. s.c.	≥0.7 MLD	hypotension, SA and AV block, but no direct chronotropic or inotropic effects
[47]	dog	into sinus node artery	up to 310 nM 3.1 μM	unchanged heart rate bradycardia (immediate slowing by 26 beats/min)
[38]	dog cat	i.v. i.v.	5 μg/kg 7 μg/kg	bradycardia of sinoatrial origin, decrease in conduction, hypotension respiratory arrest, no change or decrease in heart rate, hypotension
[35]	cat cat, dog	i.p. i.v.	1 μg/kg daily 5 μg/kg	no pathological change rapid fall in blood pressure, cessation of respiration, bradycardia, no significant ECG abnormalities, reduced contractile force
[36]	dog cat rabbit	i.v. i.v. isolated heart	5 μg/kg 5 μg/kg ~100 nM	no significant ECG abnormalities, reduced contractile force, hypotension respiratory arrest, bradycardia at otherwise unchanged ECG, hypotension no significant effect
[46]	rat	i.v.	5 μg/kg	sharp fall of blood pressure at initially unchanged heart rate and stroke volume
[43]	rat	i.v.	2.5 μg/kg 10–40 μg/kg 80 μg/kg	unchanged blood flow in the ascending aorta, hypotension and bradycardia at otherwise unchanged ECG dose-related reduction of blood flow in the ascending aorta, bradycardia appeared unrelated to the size of the TTX dose, first degree AV block, bundle brunch block, ventricular flutter/fibrillation ventricular asystole
[43]	pithed rat	i.v.	10–20 μg/kg	reduction of blood flow in the ascending aorta, hypotension, transient mild bradycardia, transient first degree AV block, bundle brunch block
[43]	rat	isolated heart	1.0 to 4.0 μg (3 μM solution)	dissociation/cessation of ventricular contractions depending on dose
[37]	cat	i.v.	1 μg/kg 2.5–10 μg/kg	unchanged heart rate, hypotension bradycardia, hypotension, slight PR prolongation, unchanged QS interval, reduced left ventricular force and reduced stroke volume
[39]	cat	i.v.	1 μg/kg	unchanged heart rate, hypotension due to a direct relaxing effect on vascular smooth muscles
[49]	cat	i.v.	1.4–3 μg/kg	prompt fall of blood pressure at unchanged heart rate and pulse pressure; initially no striking ECG alterations, increased amplitude of QRS and T wave after the development of hypotension
[45]	guinea pig	i.p.	15 μg/kg	response before respiratory arrest (≤10.3 min from injection time point): decline in blood pressure, but no change in heart rate and ECG waveform cardiac response shortly after respiratory arrest: paroxysmal ventricular tachycardia, sinus bradycardia, AV block
[48]	rat	i.a.	20 μg/kg	rapid and severe hypotension, bradycardia, heart rate increased shortly after artificial respiration was commenced
[44]	dog	i.v. (slowly)	9.3 μg/kg/hr	at apnoe: bradycardia at unchanged stroke volume, hypotension, decreased total peripheral resistance, increased pulmonary vascular resistance and increased pulmonary arterial pressure; at higher TTX concentrations (12–20 μg/kg/hr), dogs died before or shortly after apnoe, which was due to fatal hypotension

**Table 3:** Clinical grading system in tetrodotoxication according to [68].

Degree	Symptoms
First	Oral numbness and paraesthesia, sometimes accompanied by gastrointestinal symptoms (nauseaa))
Second	Numbness of face and other areas, advanced paraesthesia, motor paralysis of extremities, incoordination, slurred speech, but still normal reflexes
Third	Gross muscular incoordination, aphonia, dysphagia, dyspnoea, cyanosis, drop in blood pressure, fixed/dilated pupils, precordial pain, but victims are still conscious
Fourth	Severe respiratory failure and hypoxia, severe hypotension, bradycardia, cardiac arrhythmia, heart continues to pulsate for a short period

(a) TTX is considered as the most potent emetic agent, directly acting on the medullary chemoreceptor trigger zone [41,70].

**Table 4:** Summary on case reports between 1983 and 2009 on TTX-poisoned patients.

Study	Cases	Grade	TTX (nM)^a^	Hypotension	Sinus bradycardia	ECG	Artificial respiration	Comments
[82]	3	1–2		no	no		no	oxygen saturation 96–99%
[76,81]	4	1–2	4.5–21.1	no or mild	no	normal	no	mild hypercapnia
[83]	16	1–2					no	eight patients had hypertension
[84]	3	1–2		no			no	
[75]	177	1–3		no			no	all recovered completely
[85]	6	1–3					no	
[70]	1	2–3		yes	yes	normal^b^	no	hypoxemia, diabetes mellitus^c^
[74]	1	2–3		no	no		no	normal arterial pO_2_
[86]	4	2–3		no	intermittent^d^	normal	no	normal blood pressure, no hypoxia
[77,80]	11	2–3	<5–5 (grade 2)	no	no		yes (grade 3)	no cardiovascular effects, no fatalities
[87]	1	3		no	yes	normal	yes	reduced sensory and motor conduction velocities, decrease in evoked amplitudes
[88]	1	3		no	no	normal	yes	decreased arterial pO_2_
[76,81]	1	3	28.6	yes	no	normal	yes	
[83]	1	3–4		hypertension^c^	no		Yes (cyanosis)	diabetes mellitus^c^, renal failure and death (intoxication by molluscs)
[89]	1	4					yes	stable cardiovascular status after cardiopulmonary resuscitation; TTX-induced cranial diabetes insipidus
[65,66]	1	4	77/83	no	no	normal	yes	non-excitability of sensory and motor nerves
[85]	1	4		yes	no		yes	cardiac arrest before admission to the ED, resuscitation to sinus rhythm, patient died
[76,81]	1	4	40.6	yes	yes	AV block	yes	diabetes mellitus^c^, full cardiac arrest, spontaneous circulation after resuscitation, but patient died due to multi-organ failure
[78]	1	4	114	yes	yes	normal	yes	hypothermia, blood gases were unremarkable
[79]	1	4	164	yes			yes	complete block of motor nerve conduction
[75]	68	4		Yes (14/68)			Yes (all)	five fatalities, one patient with brain damage
[62]	83	1–4	5–43 e					seven fatal cases (respiratory arrest)
[63]	37	1–4						eight fatalities
[64]	53	1–4						eight fatalities
[90]	40	1–4						no fatalities

a Reported TTX concentrations in serum or blood samples.

b mild ST elevation.

c pre-existing disease.

d Cardiac dysautonomia did not correlate to TTX levels in blood [86].

e TTX concentrations were determined in blood or urine from 38 patients [62].
